# Sleep disturbances in bipolar disorder with comorbid post-traumatic stress disorder

**DOI:** 10.1192/j.eurpsy.2023.463

**Published:** 2023-07-19

**Authors:** F. Cruz Sanabria, C. Bonelli, D. Gravina, M. Violi, L. Massoni, S. Bruno, U. Faraguna, L. Dell’Osso, C. Carmassi

**Affiliations:** ^1^Department of Translational Research and of New Surgical and Medical Technologies; ^2^Department of Clinical and Experimental Medicine, University of Pisa, Pisa, Italy

## Abstract

**Introduction:**

Sleep disturbances are frequently reported in patients with Bipolar Disorder (BD), parallel, patients with BD report significantly higher rates of exposure to major lifetime traumatic events than the general population with a high risk of developing PTSD.

**Objectives:**

The aim of this study was to compare sleep parameters subjectively and objectively measured, in patients with BD with or without PTSD with respect to healthy control subjects.

**Methods:**

73 patients with BD (26 BD+ PTSD and 46 BDw/oPTSD) and 88 HC were evaluated through actigraphic monitoring to explore sleep and circadian parameters, scales exploring sleep quality (*Pittsburgh Sleep Quality Index -PSQI-*) and chronotype (*reduced Morningness-Eveningness Questionnaire –rMEQ-*) and the Trauma and Loss Spectrum Self Report (TALS-SR), for lifetime trauma and loss spectrum symptoms.

**Results:**

Compared to age-matched HC, patients with BD reported lower sleep quality, lower rMEQ scores suggestive of delayed chronotype, longer total sleep time, higher waking after sleep onset, lower interdaily stability and lower sleep health. Patients with BD+PTSD reported significantly higher PSQI scores than BDw/oPTSD; significant correlations between the PSQI total scores and TALS-SR symptomatic domains emerged in the BD+PTSD group only.

**Conclusions:**

Our results suggest a strong correlation between sleep disturbances, particularly evaluated by subjective measures, and PTSD symptoms in patients with BD.

**Disclosure of Interest:**

None Declared

